# Comparison of bacterial culture and 16S rRNA community profiling by clonal analysis and pyrosequencing for the characterization of the dentine caries-associated microbiome

**DOI:** 10.3389/fcimb.2014.00164

**Published:** 2014-11-12

**Authors:** Kathrin Schulze-Schweifing, Avijit Banerjee, William G. Wade

**Affiliations:** ^1^Microbiology, Dental Institute, King's College LondonLondon, UK; ^2^Conservative and MI Dentistry, Dental Institute, King's College LondonLondon, UK; ^3^Centre for Immunology and Infectious Disease, Barts and The London School of Medicine and Dentistry, Queen Mary University of LondonLondon, UK

**Keywords:** caries, oral microbiome, caries-infected dentine

## Abstract

Culture-independent analyses have greatly expanded knowledge regarding the composition of complex bacterial communities including those associated with oral diseases. A consistent finding from such studies, however, has been the under-reporting of members of the phylum *Actinobacteria*. In this study, five pairs of broad range primers targeting 16S rRNA genes were used in clonal analysis of 6 samples collected from tooth lesions involving dentine in subjects with active caries. Samples were also subjected to cultural analysis and pyrosequencing by means of the 454 platform. A diverse bacterial community of 229 species-level taxa was revealed by culture and clonal analysis, dominated by representatives of the genera *Prevotella*, *Lactobacillus*, *Selenomonas*, and *Streptococcus.* The five most abundant species were: *Lactobacillus gasseri*, *Prevotella denticola*, *Alloprevotella tannerae*, *S. mutans* and *Streptococcus* sp. HOT 070, which together made up 31.6 % of the sequences. Two samples were dominated by lactobacilli, while the remaining samples had low numbers of lactobacilli but significantly higher numbers of *Prevotella* species. The different primer pairs produced broadly similar data but proportions of the phylum *Bacteroidetes* were significantly higher when primer 1387R was used. All of the primer sets underestimated the proportion of *Actinobacteria* compared to culture. Pyrosequencing analysis of the samples was performed to a depth of sequencing of 4293 sequences per sample which were identified to 264 species-level taxa, and resulted in significantly higher coverage estimates than the clonal analysis. Pyrosequencing, however, also underestimated the relative abundance of *Actinobacteria* compared to culture.

## Introduction

Dental caries, or tooth decay, is the dissolution of tooth structure by acids formed by bacteria as a result of the fermentation of dietary carbohydrate, particularly sucrose. *Streptococcus mutans* was one of the first species associated with dental decay leading to carious lesions in teeth (Clarke, 1924; Loesche et al., 1975). *S. mutans*-free caries lesions have been observed, however, (Marsh et al., 1989) and it has been recognized that the plaque biofilm as a whole, rather than individual species, is responsible for acid production and lesion formation, particularly in the early stages (Marsh, 2003; Takahashi and Nyvad, 2011). Consequently, it is important to comprehensively determine the composition of the bacterial community associated with dental caries to better understand the bacterial factors and host and environmental interactions that are responsible for the initiation and progression of dental decay. This will make it possible to develop novel preventative and/or therapeutic strategies for this disease.

Traditionally, microbiologists have used culture media to grow and characterize bacterial species, but it has been realized that not all species can be readily grown under laboratory conditions. Consequently, in recent years molecular methods targeting the 16S rRNA gene to characterize complex microbial communities have been established and many sequences representing novel species have been detected. There are around 700 bacterial species found in the human mouth, around 65% of which have been cultured (Paster et al., 2001, 2006; Dewhirst et al., 2010).

The culture-independent methods themselves have biases and deficiencies; for example it has been found that the proportions of *Actinobacteria* were underestimated using molecular analysis when a direct comparison to culture was available (Munson et al., 2002, 2004; de Lillo et al., 2006). Furthermore, recent studies by Tanner et al. (2011) and Kanasi et al. (2010) observed greater diversity of species detected in early childhood caries (ECC) using culture compared to clonal analysis.

The caries-associated microbiota has yet to be completely characterized; many recent studies in have reported the detection of novel species, genera or even higher taxonomic orders (Munson et al., 2004; Nadkarni et al., 2004; Chhour et al., 2005; Kanasi et al., 2010; Tanner et al., 2011). Tanner et al. (2011) reported *Scardovia wiggsiae* to be significantly associated with severe ECC children in the presence and absence of *S. mutans* detection and showed for the first time a strong association of *S. wiggsiae* together with *S. mutans* in ECC. These findings clearly demonstrate that continued efforts to characterize the microbiota of caries and distinguish mechanisms of disease progression are needed.

The aim of this study was firstly, to design novel primers for 16S rRNA-based community profiling of the microbiota associated with carious dentine, and, secondly, to compare the results obtained with cultural and pyrosequencing analyses of the same samples.

## Materials and methods

### Subjects and sample collection

Ethical approval for the study was granted by the Lewisham Local Research Ethics Committee South London REC Office (4) (Reference 08/H0810/61). Six subjects, four male and two female, aged 22–35 years (mean age 26.6 years), who were medically healthy participated in the study with their informed consent, provided in writing. Subjects were included if they had a carious lesion that had spread into the middle or inner third of dentine, that was checked radiographically with cavitation. Local anesthesia was administered where necessary, and the carious teeth isolated with rubber dam to minimize saliva contamination during the excavation procedure. Following removal of carious enamel to the enamel-dentine junction with a sterile, water-cooled diamond bur in an air-turbine handpiece, the dentine lesion was hand excavated with a sterile, spoon excavator (Ash G5; Claudius Ash Ltd., Potters Bar, UK). After the superficial layer of debris had been removed and discarded, the sample, consisting of soft necrotic dentine, was collected using a fresh, sterile spoon excavator at a level that represented the infected dentine lesion. Samples were placed in 1 ml of reduced transport medium (1% w/v tryptone, 0.5% w/v yeast extract, 0.1% w/v L-cysteine, 0.1% w/v D+glucose, 2% v/v horse serum in distilled water and adjusted to pH 7.5, RTM). Samples were then vortex-mixed for 1 min and then divided.

### Bacterial culture

Ten-fold serial dilutions of 100 μl of the sample suspensions were prepared in RTM within an anaerobic workstation. One hundred μl of appropriate dilutions were used to inoculate pre-reduced Fastidious Anaerobe Agar (LabM, Bury, UK) +5% horse blood (FAA) plates, in triplicate, which were incubated anaerobically for 10 d at 37°C. Plates with between 30 and 300 colonies were counted and 96 colonies were selected randomly and subcultured on FAA plates, with a *Propionibacterium acnes* feeder streak. Isolates were incubated anaerobically for a further 4–5 days, after which the purity of all isolates was visually checked using a plate microscope. Mixed cultures were subcultured to achieve purity and pure cultures were stored at −70°C in Brain Heart Infusion (BHI) +10% glycerol. Cells were harvested from FAA plates of the isolates, and suspended in 1 ml PBS (Oxoid). DNA was extracted by means of the GenElute™ bacterial genomic kit (Sigma Aldrich), following the modification for Gram-positive bacteria. 16S rRNA genes were amplified by PCR with primer pair 27F CM/1492R. Reactions were prepared containing 4 μl 5× Phusion buffer GC, 0.4 μl 10 mM dNTPs, 0.2 μl Phusion HF polymerase (0.4 U, Finnzymes), 0.5 μl of each primer (10 μM), 1 μl of template and 13.4 μl sterile water. Initial denaturation was at 98°C for 30 s, followed by 30 cycles of denaturation at 98°C for 10 s, annealing at 56°C for 30 s and extension at 72°C for 45 s.

### PCR primer design

The Human Oral Microbiome Database (Chen et al., 2010) reference dataset was aligned and manually inspected for regions of homology suitable for PCR primer design. Novel primer 39F (Table 1) was selected for use in this study. Primer 61F was modified from primer 63F described by Marchesi et al. (1998) and was truncated at the 3′ end to remove the mis-match with many oral streptococcal species (Table 1).

**Table 1:** **Primers used in the study**.

**Primer**	**Sequence (5′-3′)**	
27F CM	AGAGTTTGATCMTGGCTCAG	Lane, 1991
27F YM	AGAGTTTGATYMTGGCTCAG	Frank et al., 2008
39F	ATCMTGGCTCAGRWYGAACGC	This study
61F	CAGGCCTAACACATGCAAG	This study
519R	GWATTACCGCGGCKGCTG	Lane, 1991
1387R	GGGCGGWGTGTACAAGGC	Marchesi et al., 1998
1492R	TACGGYTACCTTGTTACGACTT	Lane, 1991

### Community profiling by clonal analysis

The remaining 900 μl of the original samples was centrifuged for 10 min at 13,000 g, the supernatant discarded and the pellet subsequently used for DNA extraction using the GenElute™ bacterial genomic kit. 16S rRNA genes of the extracted DNA from each patient sample were amplified with the following primer combinations: 27F YM/1492R (library 1), 27F CM/1492R (library 2), 39F/1387R (library 3), 39F/1492R (library 4) and 61F/1387R (library 5) (Table 1). Five replicate amplification reactions were set up for each sample and combined. Initial denaturation was at 95°C for 5 min, followed by 25 cycles of denaturation at 95°C for 45 s, annealing at 50°C for 45 s and extension at 72°C for 90 s. Ten μl of the five replicate *Taq* polymerase PCR products of each primer set were pooled and cloned into the TA cloning vector pCR4-TOPO (Invitrogen) following the manufacturer's instructions. Transformants were detected on LB agar supplemented with 50 μg/ml kanamycin. Ninety-six clone colonies were chosen at random and the insert amplified by PCR with vector-specific primers M13 FWD and REV.

#### Sequencing

PCR products from clone insert amplification or from isolates amplified with Phusion polymerase were purified using the ExoSAP-IT (Exonuclease I/Shrimp Alkaline Phosphatase) clean up kit (Affymetrix, High Wycombe, UK). Five μl of PCR product were mixed with 1 μl ExoSAP-IT and 1 μl water and incubated in a thermal cycler for 15 min at 37°C followed by incubation at 80°C for 15 min. For each sample, 96 isolates and 96 clones from each library were partially sequenced using primer 519R. Reactions were set up with 0.5 μl BigDye (Applied Biosystems, Life Technologies), 1.75 μl 5 × sequencing buffer (Applied Biosystems), 0.3 μl primer 519R (10 μM), 5.45 μl deionised, autoclaved water and 2 μl cleaned up PCR product as template. Thirty cycles were run consisting of 10 s at 96°C, 5 s at 50°C and 2 min at 60°C. Sequencing reaction products were purified by ethanol precipitation and then dissolved in 10 μl 0.1 × TE and sequenced by means of an AB3730xl DNA analyser (Applied Biosystems). Sequences were identified by BLASTn interrogation of the Human Oral Microbiome Database (HOMD), by means of the HOMD BLAST on-line tool (www.homd.org). The following parameters were used with BLASTn: cost to open a gap = 5; cost to extend a gap = 5; penalty for a mismatch in the blast portion of run = −5; reward for a match in the blast portion of run = 4. A word length of 11 was used. Sequences were compared with database sequences at a sequence identity level of 98.5%, and greater than 90% shared coverage for positive identification. Sequences with multiple database hits abover 98.5% were reported with all possible identification options.

### Pyrosequencing

For amplicon library construction, 16S rRNA genes of the DNA extracted from the samples from the initial patient sample were amplified using six barcoded forward primers which consisted of the 27FYM template-specific primer sequence, a 12-base unique Golay barcode and the Lib-L Adapator A. The reverse primer consisted of template-specific reverse primer 519R sequence with Lib-L Adapator B. Three replicate amplification reactions were set up for each sample. Reactions were prepared containing 12.5 μl Extensor PCR mastermix (High fidelity Taq polymerase, Thermo Scientific), 2 μl of template, 0.5 μl of each primer (10 μM) and 9.5 μl sterile water. Initial denaturation was at 95°C for 5 min, followed by 25 cycles of denaturation at 95°C for 45 s, annealing at 53°C for 45 s, extension at 72°C for 90 s and a final extension at 72°C for 15 s. PCR amplicons were pooled and then purified using the QIAquick PCR purification kit (Qiagen, Crawley, UK) following the manufacturer's instructions. The size and purity of the amplicons were assessed using the Agilent 2100 Bioanalyzer and the Agilent DNA 1000 kit (Agilent Technologies, Inc., Wokingham, UK), and quantified using the Quant-iT-Picogreen fluorescent nucleic acid stain (Invitrogen). The amplicons were then pooled at an equimolar concentration of 1 × 10^9^ molecules/μl. The pooled samples were amplified clonally by emulsion-PCR using the GS emPCR Lib-L Kit. The GS PicoTiterPlate Kit was then used to sequence individual clonally amplified molecules on a Roche 454 GS-FLX Titanium sequencer. The data were analyzed using the mothur pipeline (Schloss et al., 2009). Trim.flows was used to remove sequences with more than two mismatches to the primer sequences or more than one mismatch to the barcode and sequences with fewer than 320 or greater than 720 flows. Sequences were de-noised by means of shhh.flows after which trim.seqs was used to remove the primer and barcode sequences, and sequences shorter than 350 bp. The sequences were then aligned to the Silva reference file by means of align.seqs, after which non-aligned sequences and columns without bases were removed. Pre.cluster was used to combine sequences that were within 1 bp per 100 bp of total sequence length of a more abundant sequence with that sequence. Chimerae were detected using chimera.uchime and removed using remove.seqs. The classify.seqs command was used to classify sequences using the HOMD version 10 reference sequence and taxonomy databases. The dist.seqs program calculated uncorrected pairwise distances between aligned sequences and the cluster command was used to assign sequences to OTUs. Following this, a table was created indicating the number of times an OTU was present in each sample using the make.shared command. Because the groups for the different patients contained varying amounts of sequences, all samples were normalized to the size of the smallest sample group (4293 sequences) by means of sub.sample. The classify.otu command was used to obtain a consensus taxonomy for each OTU. The collect.single command was used to calculate the Chao1 richness and the Inverse Simpson diversity index. A table containing the number of sequences, sample coverage, number of observed OTUs and the Inverse Simpson diversity estimate was compiled using the summary.single command.

The pyrosequencing data was deposited in the NCBI SRA database as accession SRP047474.

### Statistical analysis

The relative abundance of taxa in samples and by different analysis methods was compared by means of a two tailed *Z*-Test calculator for paired comparisons, with a significance threshold of 0.05.

## Results

### Culture and 16S rRNA gene sequencing and cloning analysis

The mean bacterial count estimated from culture on the anaerobically FAA agar was 6.5 × 10^7^, and ranged from 5.4 × 10^4^ to 2.0 × 10^8^. The sequences from 2700 cloned 16S rRNA genes and 540 isolates were analyzed together. 229 taxa were found at species level, representing 8 phyla: *Firmicutes*, *Proteobacteria*, *Actinobacteria*, *Bacteroidetes*, *Fusobacteria*, TM7, *Spirochaetes* and *Synergistetes*. 216 taxa were detected using molecular analysis, of which 143 were only found using this method. 86 taxa were isolated in culture, 12 of which were not detected using molecular analysis. Sequences representing 16 novel taxa were identified and were sequenced to near full length and the sequences deposited in the NCBI nucleotide database (Supplementary Table 1).

The distribution of taxa found in the dentine caries lesions detected by culture and clonal analysis is shown in Supplementary Table 2. A highly taxon-rich community was seen, including numerous representatives of the genera *Prevotella*, *Lactobacillus*, *Selenomonas*, and *Streptococcus.* The five most abundant species overall were *Lactobacillus gasseri*, *Prevotella denticola*, *Alloprevotella tannerae*, *S. mutans* and *Streptococcus* sp. HOT 070, who together represented 31.6% of the total sequences. There were differences in the composition of the microbiota in samples from different subjects; e.g., lactobacilli made up 93.2 and 57.6% of the microbiota in samples A and E, respectively but only 0.2 and 1.5% in B and D, while none were detected in patients C and F (*p* < 0.05). Few other *Firmicutes* were observed in subject A, whilst the sample from subject E showed a significantly higher proportion of *S. mutans* and *Veillonella dispar/parvula* (*p* < 0.05). Samples from subjects B, C, D, and F had a wide range of other *Firmicutes*.

Subject C had significantly higher levels of *Atopobium rimae* and *Atopobium* OT416 compared to all other subjects (*p* < 0.05) and 88 of the detected 92 *Pseudoramibacter alactolyticus* clones were detected in this sample (*p* < 0.05). Compared to the levels of lactobacilli seen, significantly fewer *Prevotella* species were detected in samples A and E (*p* < 0.05). *Prevotella* spp. comprised between 21.1 and 40.2% of the microbiota in samples B, C, D, and F, which was significantly higher than that found in samples A and E. Of the 57 *Olsenella profusa* sequences detected, 19 and 37 were detected in isolate libraries of samples D and F, respectively, which was significantly higher than was found in the molecular libraries of these patients as well as in the remaining subjects (*p* < 0.05). Spirochetes and TM7 were both detected in patient B and D, but the relative occurrence was reversed; i.e., patient B had significantly more TM7 than *Spirochaetes* (*p* < 0.05), while patient D had significantly more of the latter and only few TM7 (*p* < 0.05).

**Figure 1:**
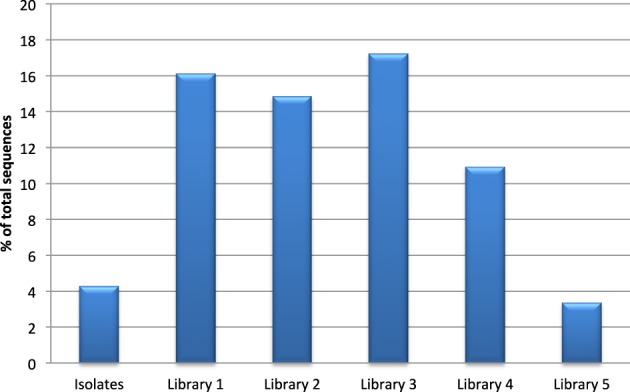
**Detection of the genus *Streptococcus* by culture and clonal analysis**.

All clone libraries significantly under-reported *Actinobacteria* numbers compared to culture analysis (*p* < 0.05). The detection of *Bacteroidetes* seemed to be influenced mostly by the choice of reverse primer as libraries 3 and 5 that used primer 1387R had a significantly higher rate of detection of *Bacteroidetes* (*p* < 0.05) than the libraries/culture analysis using primer 1492R. The highest detection of *Firmicutes* was seen in the libraries where 27F CM was used. The proportion of the microbiota represented by the genus *Streptococcus* was significantly higher in libraries 1, 2, 3, and 4 compared to culture (*p* < 0.05, Figure 1). Streptococcal proportions in library 5, were not significantly different to those revealed by culture, but there was a significant difference compared to the other molecular libraries (*p* < 0.05).

**Figure 2:**
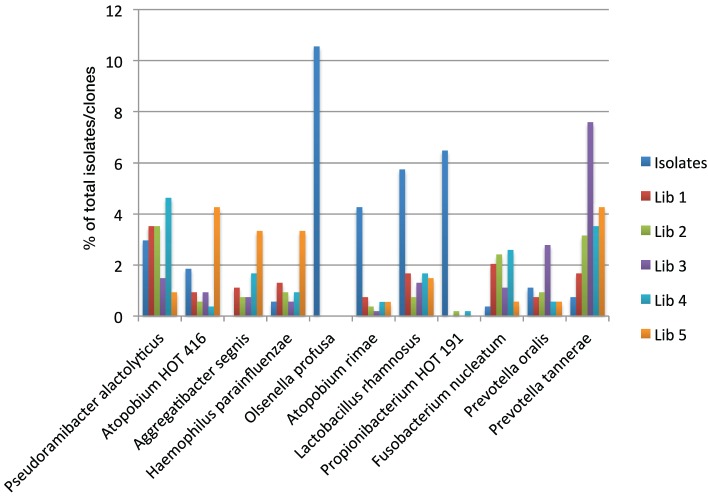
**Detection of selected oral taxa by culture and clonal analysis**.

Figure 2 shows the proportions of selected taxa among the isolates and clones. The taxa shown were chosen because the total number of sequences detected for each taxon was greater than 1% of the total and at least one library showed an at least 50% higher incidence compared to the other libraries or detection rates in two libraries was at most 50% of the detection rate of the other libraries. For example, libraries prepared with reverse primer 1387R (3 and 5) detected between 50 and 75% less *Pseudoramibacter alactolyticus* compared to reverse primer 1492R (*p* < 0.05). Detection of *Atopobium* OT 416, *Aggregatibacter segnis* and *Haemophilus parainfluenzae* was significantly increased with primer pair 61F/1387R (library 5) to more than double of that detected with culture analysis or any other primer pair combination used (*p* < 0.05). *Olsenella profusa* was only found using culture methods (*p* < 0.05). Detection of *Atopobium rimae*, *Lactobacillus rhamnosus*, and *Propionibacterium* OT 191 showed a similar trend in that detection using culture was on average 11.0, 5.5, and 34.1 times higher than that using molecular methods, respectively (*p* < 0.05). Detection of *Fusobacteria* was significantly reduced when culture methods or reverse primer 1387R (libraries 3 and 5) were used; detection of *Prevotella oralis* and *Prevotella tannerae* was significantly increased using primer pair 39F/1387R (library 3) (*p* < 0.05).

### Pyrosequencing

35,191 sequences were obtained by pyrosequencing. After filtering for quality and removal of chimerae, 29,835 sequences were analyzed. The number of sequences per sample ranged from 4293 to 6488 and thus the sample libraries were sub-sampled to select 4293 sequences from each sample.

Table 2 shows a comparison of the OTU-based analysis of the clonal data obtained with library 2 (primer set 27FYM/1492R) and pyrosequencing. The number of sequences obtained from pyrosequencing was almost 50-fold higher, whilst the number of observed OTUs (S_obs_) was between 4.05 and 7.44 times greater in 454 sequencing. Chao1 estimates of total species richness were 2 and 14 times higher for pyrosequencing. Good's coverage estimates were 97% or higher for pyrosequencing but generally substantially lower for the clonal analysis, ranging from 67 to 97%.

**Table 2:** **Comparison of coverage and taxon richness estimates by clonal analysis and pyrosequencing**.

**Analysis sample**	**Clonal analysis—Library 2 (*n* = 90)**			**Pyrosequencing (*n* = 4293)**		
	**Sobs**	**Good's coverage**	**Chao1**	**Sobs**	**Good's coverage**	**Chao1**
A	9	0.97	10	67	0.99	142.6
B	51	0.67	73.56	331	0.97	486.22
C	25	0.88	34.17	176	0.98	282.64
D	42	0.73	69.6	287	0.97	441.89
E	19	0.84	64.5	77	0.99	121
F	39	0.73	94.2	187	0.98	263.24

The distribution of phyla in the samples seen in the pyrosequencing analysis was broadly similar to that revealed by cloning and Sanger sequencing. The detection of *Actinobacteria* was significantly lower than that seen by culture (*p* < 0.05). Eighty seven genera were detected by pyrosequencing, 65 of which had been found by Sanger sequencing, whilst 25 were only detected by pyrosequencing. Four genera were found only with Sanger sequencing. Of the 25 genera found only by pyrosequencing most (17 of 25) were represented by five or fewer sequences.

The identification of the sequences to species level using the mothur command classify.seqs in conjunction with the HOMD reference dataset is shown in Supplementary Table 3. A bootstrap cut-off value of 80% was used. Unclassified sequences were those that did not match any database sequence or where two closely related species could not be differentiated. 264 species-level taxa were detected by pyrosequencing, including numerous representatives of the genera *Prevotella*, *Lactobacillus*, *Streptococcus*, *P. alactolyticus*, and *Fusobacteria.* The five most abundant species-level taxa were *Lactobacillus gasseri*, a group of unclassified streptococci, *S. mutans*, *P. alactolyticus*, and *P. denticola*; together representing 40.7% of all detected sequences. Three of the five most abundant species were the same as for Sanger sequencing: *L. gasseri*, *S. mutans*, and *P. denticola*, but their proportions were significantly different (*p* < 0.05).

Differences in the composition of the microbiota in samples from different subjects were observed as seen in the Sanger sequencing analysis and the same proportional trends were observed for the pyrosequencing data. For example, both sequencing methods showed that samples A and E had significantly higher proportion of lactobacilli than the other patients, with few other *Firmicutes* observed in patient A, whereas sample E showed a high proportion of *S. mutans* and *Veillonella parvula* (*p* < 0.05). Similarly, pyrosequencing confirmed the results of the Sanger analysis in that *Prevotella* species were detected at low levels in patient samples A and E (0–0.32% of sequences), while making up a substantial proportion of the microbiota in samples B (14.2%), C (43.5%), D (37.1%) and F (11.9%).

## Discussion

One of the primary aims of this study was to improve the detection of high G+C species in the molecular analyses. This was not achieved and the results were similar to those from previous studies (Munson et al., 2002, 2004; de Lillo et al., 2006), in that the proportions of *Actinobacteria* in the molecular analyses were reduced compared to culture, although primer pair 61F/1387R was superior to the other primer pairs tested. The different primers in their various combinations used for the molecular analysis of the carious samples resulted in different, but minor biases in the detection of various species. Although the primers used to target 16S rRNA genes in molecular ecology studies are often referred to as “universal” primers, it would be more appropriate for them to be termed broad range primers, since there is sequence variation in even the conserved regions of the gene (Wang et al., 2009; Palatinszky et al., 2011). Furthermore, primers have been designed using alignments of species known to date, which frequently contain sequencing errors (Taylor et al., 2007). Single mismatches in the first 3–4 nucleotides from the 3′ end of a PVR primer can greatly reduce and even hinder extension (Bru et al., 2008; Wu et al., 2009). Bru et al. (2008) reported differences in the detection rates using forward and reverse primers with a single mismatch, which could explain the observed differences in detection for the various primers. They found forward primers with a mismatch located more than four bases away from the 3′ end underestimated gene copy numbers, while no effect was seen on the reverse primers. They conceded that the severity of this bias is determined by numerous factors, such as the primer length, the nature, and position of the mismatches and the annealing temperature of the primers. Overall, it appears that the choice of primers cannot explain the bias against members of the phylum *Actinobacteria*.

A potential source of bias is the cloning used in the clonal analysis. It has been found that libraries from an environmental sample constructed using the TA-cloning of multi-template PCR products showed significant differences compared to a length heterogeneity PCR which did not employ cloning (Palatinszky et al., 2011). However, Taylor et al. (2007) found no biases due to TA cloning when testing phylogenetic bias in fungal environmental clone library construction comparing TA and blunt-ended cloning. The abundance of OTUs was found to be correlated and phylogenetic tests showed no significant differences between the two libraries (Taylor et al., 2007). Some OTUs differed in abundance between libraries, however, indicating a potential phylogenetic bias during cloning. Palatinszky et al. (2011) suggested that clone libraries with low diversity may be more prone to phylogenetic biases and that drawing conclusions from diverse communities such as used by Taylor et al. (2007) could lead to underestimation of the extent of the bias.

When comparing the distribution of taxa of all libraries combined for each patient it became apparent that when a patient has a high incidence of lactobacilli (patients A and E), few *Prevotella* species (A = 1, E = 8 sequences identified as *Prevotella*) and no *Pseudoramibacter* were observed and vice versa (patient F = 8 sequences identified as lactobacilli). Furthermore, where the microbiota of two samples was dominated by lactobacilli the overall number of taxa was lower compared to the other samples (22 and 41 taxa vs. 113, 60, 117, and 88 taxa). Chhour et al. (2005) reported similar findings when looking at the microbial diversity in advanced caries lesions of 10 patients. They, too, reported *Lactobacillaceae* and *Prevotellaceae* to make up the majority of all identified sequences and that lesions could be grouped into *Prevotella*-dominated or *Lactobacillus*-dominated samples. They argued that colonization, or exclusion thereof, could depend on fermentation by-products, but that lactate did not appear to be the major fermentation by-product, since they observed lactate-dependent *Veillonella* spp. infrequently and no further species capable of metabolizing lactate. Similar results were found here, particularly that lactate-utilizing phylotypes, such as *Selenomonas* spp. and *Pseudoramibacter alactolyticus*, were detected in lesions high in *Prevotella* (Chhour et al., 2005). The conclusion from these observations are that the incidence of the dominant species depends on factors early in the colonization of the dentine matrix and successive inclusion or exclusion of subsequent colonizers is likely to be determined by metabolic by-products of the initial colonizers (Byun et al., 2004; Nadkarni et al., 2004; Chhour et al., 2005).

Another approach to advance the detection of novel species could be to use the universal primers to detect new groups of bacteria, as was achieved in this study, and to subsequently design family/genus-specific primers to broaden the knowledge of that particular group. It was shown in several other studies that greater diversity could be observed using genus specific primers (Vartoukian et al., 2009; Lin et al., 2011; Xie et al., 2011).

As previously mentioned, this study, employing five primer sets (some of them novel), resulted in similar findings to other studies looking at the microbiota of dentine caries. Munson et al. (2004) analysing the microbiota of five carious dentine samples found 95 taxa when applying two primer sets and culture analysis. They found three taxa to be detectable in all five samples and found *O. profusa* and *P. acidifaciens* to be predominant in culture analysis, but very few clones could be identified as these two species. In this study, a total of 228 taxa were found of which only a single taxon, *S. mitis/pneumonia*e, was found in all six patient samples. Similar to the above mentioned study, *O. profusa*, *O. uli*, and *P. acnes* were predominant in culture analysis, but none or only a few were detected in the molecular analysis. These findings are in contrast to those by Chhour et al. (2005) who, analysing ten carious dentine samples using primer pair 331F/797R, found *O. profusa* and *P. acidifaciens* to make up to 14.4 % and 30 % of the detected bacterial load, respectively. Studies by Tanner et al. (2011) and Kanasi et al. (2010) looked at culture and molecular analysis of the same plaque samples taken from children with and without early childhood caries (ECC). The culture-based study strongly associated *S. mutans* with disease, but also found a diverse microbiota as well as a novel potential pathogen associated with ECC, *Scardovia wiggsiae* (Tanner et al., 2011). Kanasi et al. (2010), analysing the same sample pool using molecular techniques reported 139 taxa (and 35 provisional taxa) and found *S. mutans* and *Bifidobacteriaceae* to be significantly associated with ECC. However, this was only discovered using specific PCR primers, indicating that caries sites are highly diverse and that, while important in disease, the organisms may be present only in low proportions. Another explanation could of course be that the universal primers are not specific enough to allow for full detection of those species. In this study, *S. mutans* was detected in five of the six patient samples using culture and molecular techniques, albeit in varying frequencies. These results suggest that the combination of some patients having low numbers of this species and the choice of primers greatly influences rate of detection. Overall, had only primer pair 27F YM/1492R been used 62 taxa that were detected in this study would have been overlooked. Even using primer pair 27F CM/1492R, that allowed detection of the most taxa not found with any other primer combination, would result in 51 taxa not being detected.

Similarly, if no culture analysis had been performed, none of the 57 *O. profusa* isolates would have been detected and thereby this taxon would have been completely missed in the clonal analysis, although it was detected by pyrosequencing. It seems less likely that novel species will be detected using established culture methods. Tanner et al. (2011), however, by using careful sample handling and multiple culture media, isolated 45 previously uncultivated taxa and 45 potentially novel species-level taxa not in the HOMD database.

It was clear that the use of pyrosequencing and its inherent increased depth of analysis did increase coverage compared to clonal analysis, especially in taxon-rich samples, such as B and D. Pyrosequencing also helped enhance knowledge of the microbiota in dentine caries, in that a number of genera were found, which were not seen with Sanger sequencing. Most of those sequences were detected in low abundances and may represent part of the rare biosphere (Pedros-Alio, 2012). This can be explained by the high number of sequences generated by pyrosequencing, whereby the chance of detecting a rare species is increased. Indeed, two sequences belonging to the phylum SR1 and one sequence belonging to the phylum *Chloroflexi* were detected that have previously been described as rare (Keijser et al., 2008). Diaz et al. (2012) argued that OTUs representing singletons should be eliminated, since it was found that in communities with known numbers of OTUs pyrosequencing and following analysis generated more OTUs than expected. However, findings from this study show that OTUs from the rare biosphere would thus be missed and steps to eliminate or include OTUs in the analysis have to be given careful consideration (Reeder and Knight, 2009; Schloss and Westcott, 2011).

In previous studies, detection of *Veillonella* spp. was reported in most patients at all stages ranging from intact to deep dentine cavities, but no significance could be drawn in relation to any stage of caries lesion progression (Aas et al., 2008; Gross et al., 2010; Lima et al., 2011), showing that the significance of the detection of certain species and genera is still not clear. When Belda-Ferre et al. (2012) made comparisons at species level between health and caries status, however, *Veillonella parvula* was found in caries-active and caries-free patients. Only the metagenomic analysis showed that different strains were present in health and disease, since the *Veillonella* found in caries-active individuals contained genes that *Veillonella* in caries-free subjects did not, which could indicate that different genes are involved in pathogenesis (Belda-Ferre et al., 2012). Metagenomic analyses which take into account genetic variation among strains within a species and metatranscriptomic methods which determine which genes are actively transcribed clearly have advantages over 16S rRNA-based analyses in determining the function of the oral microbiome in health and disease.

### Conflict of interest statement

The authors declare that the research was conducted in the absence of any commercial or financial relationships that could be construed as a potential conflict of interest.

## Supplementary material

Supplementary Table 1 **Accession number of 16S rRNA gene sequences for novel taxa identified in the study**.

Supplementary Table 2 **Caries-associated bacterial biota determined by culture and clonal analysis**. Library 1 – primers 27F CM/1492R; Library 2 – 27F YM/1492R; Library 3 – 39F/1387R; Library 4 - 39F/1492R; Library 5 – 61F/1387R.

Supplementary Table 3 **Caries-associated bacterial biota determined by pyrosequencing**.
