# Supplementary material for: Comparison of bacterial culture and 16S rRNA community profiling by clonal analysis and pyrosequencing for the characterization of the dentine caries-associated microbiome
Source: Front Cell Infect Microbiol. 2014 Nov 12;4:164. doi: 10.3389/fcimb.2014.00164 (PMC4228914; doi:10.3389/fcimb.2014.00164)
Supplement: Supplementary Table 1 — Accession number of 16S rRNA gene sequences for novel taxa identified in the study. [file Table1.DOCX]

**Table 1. Novel taxa detected in carious lesions**

| Clone no. | Study identification | HOMD oral taxon no. | Accession number |
| --- | --- | --- | --- |
| AL159 | *Haemophilus* K1 | 908 | JQ406529 |
| BL117 | *Ottowia* K1 | 894 | JQ406530 |
| BL163 | *Erysipelotrichaceae* K1 | 904 | JQ406531 |
| BL176 | *Alloprevotella* K1 | 912 | JQ406532 |
| BL190 | *Bergeyella* K1 | 907 | JQ406533 |
| BL216 | *Alloprevotella* K2 | 913 | JQ406534 |
| BL218 | *Capnocytophaga* K1 | 901 | JQ406535 |
| BL234 | *Bacteroidales* K1 | 899 | JQ406536 |
| DL111 | *Veillonellaceae* K1 | 918 | JQ406537 |
| DL151 | *Erysipelotrichaceae* K2 | 905 | JQ406538 |
| DL303 | *Alloprevotella* K3 | 914 | JQ406539 |
| DL325 | *Bacteroidales* K1 | 911 | JQ406540 |
| EL130 | *Veillonella* K1 | 917 | JQ406541 |
| FL163 | *Capnocytophaga* K2 | 902 | JQ406542 |
| FL377 | *Actinomyces* K1 | 896 | JQ406543 |
| CL133 | *Moryella* K1 | 910 | JQ406544 |
